# Menin is necessary for long term maintenance of meningioma-1 driven leukemia

**DOI:** 10.1038/s41375-021-01146-z

**Published:** 2021-02-04

**Authors:** Clara Libbrecht, Hongbo M. Xie, Molly C. Kingsley, Jessica N. Haladyna, Simone S. Riedel, Fatemeh Alikarami, Alexandra Lenard, Gerard M. McGeehan, Patricia Ernst, Kathrin M. Bernt

**Affiliations:** 1 Division of Pediatric Oncology, Children’s Hospital of Philadelphia, Philadelphia, PA USA; 2 Institut d’Hématologie et d’Oncologie Pédiatrique, Lyon, France; 3 Department of Bioinformatics and Health Informatics (DBHI), Children’s Hospital of Philadelphia, Philadelphia, PA USA; 4 Department of Pediatrics, Section of Hematology/Oncology/BMT, University of Colorado, Denver/Anschutz Medical Campus, Aurora, CO USA; 5 Syndax Pharmaceuticals, Inc, Waltham, PA USA; 6 Department of Pharmacology, University of Colorado, Denver/Anschutz Medical Campus, Aurora, CO USA; 7 Department of Pediatrics, Perelman School of Medicine at the University of Pennsylvania and Abramson Cancer Center, Philadelphia, PA USA; 8 Division of Oncology and Center for Childhood Cancer Research, Children’s Hospital of Philadelphia, 3501 Civic Center Boulevard, CTRB 3064, Philadelphia, PA 19104 USA

**Keywords:** Oncogenesis, Acute myeloid leukaemia

## Abstract

Translocations of *Meningioma-1 (MN1)* occur in a subset of acute myeloid leukemias (AML) and result in high expression of MN1, either as a full-length protein, or as a fusion protein that includes most of the N-terminus of MN1. High levels of MN1 correlate with poor prognosis. When overexpressed in murine hematopoietic progenitors, MN1 causes an aggressive AML characterized by an aberrant myeloid precursor-like gene expression program that shares features of *KMT2A*-rearranged (*KMT2A*-r) leukemia, including high levels of *Hoxa* and *Meis1* gene expression. Compounds that target a critical KMT2A–Menin interaction have proven effective in *KMT2A*-r leukemia. Here, we demonstrate that Menin (*Men1*) is also critical for the self-renewal of MN1-driven AML through the maintenance of a distinct gene expression program. Genetic inactivation of *Men1* led to a decrease in the number of functional leukemia-initiating cells. Pharmacologic inhibition of the KMT2A–Menin interaction decreased colony-forming activity, induced differentiation programs in MN1-driven murine leukemia and decreased leukemic burden in a human AML xenograft carrying an *MN1-ETV6* translocation. Collectively, these results nominate Menin inhibition as a promising therapeutic strategy in MN1-driven leukemia.

## Introduction

Acute myeloid leukemia (AML) is a heterogenous disease characterized by the clonal proliferation of myeloid cells arrested at an early stage of differentiation. Genome-wide sequencing efforts have allowed the identification of a large number of molecular abnormalities [1, 2], but for the most part, the exact contribution of each abnormality to leukemogenesis remains unclear.

The overexpression of Meningioma-1 (MN1) is common in AML. High expression levels of MN1 have been shown to correlate with poor prognosis [3–9], with the exception of leukemias characterized by the presence of an inv(16) [9, 10]. Less commonly, the *MN1* gene is involved in translocations with the genes encoding the transcription factors ETV6 [4, 11], FLI1 [12, 13], or STAT3 [4]. Furthermore, rearrangements of the *MN1* locus were recently described that result in high expression of full-length MN1 presumably via enhancer hijacking [4]. Side by side comparison of MN1 and MN1-FLI1 overexpression showed that the FLI1 protein partner is not required for transformation but confers a megakaryoblastic phenotype [13].

MN1 remains poorly characterized both functionally and structurally. Some evidence suggests that MN1 can act as transcriptional activator [14, 15]. Forced expression of MN1 in murine bone marrow causes the development of an aggressive leukemia. Early myeloid progenitors were found to be particularly susceptible to MN1 overexpression as their high level of *Hoxa9* and *Meis1* expression is a transcriptional requirement for MN1-mediated transformation [16]. MN1-driven leukemic cells harbor an aberrant gene expression program characterized by genes that are normally downregulated at the transition from common myeloid progenitors (CMP) to granulocytes macrophages progenitors, such as *Hoxa9* and *Meis1* [16]. A similar gene expression requirement exists in human CD34 + cord blood cells, where MN1 alone is insufficient to induce leukemia but needs cooperation with high *HOX* cluster gene expression mediated for example by the *NUP98-HOXD13* fusion [17].

*HOX* genes and their co-factor *MEIS1* are target genes of both KMT2A-fusions and wild-type KMT2A proteins [18]. We previously found that loss of *Kmt2a* led to downregulation of the aberrant “MN1-driven leukemic program”, suggesting that the aberrant expression of *Hoxa9* and *Meis1* in MN1-driven leukemia remained under the control of its physiologic regulator, Kmt2a [19].

KMT2A, the human homolog of the Drosophila Trithorax [20], can target chromatin through multiple distinct domains. The KMT2A C-terminus contains a conserved Su(var)3–9, enhancer of zeste trithorax (SET) domain, involved in methylation of lysine 4 at histone 3 (H3K4me) [21, 22]. The N-terminal region contains DNA-binding domains: three AT hooks [23] and a CxxC domain [24]. The N-terminus also contains a binding domain for Menin, a scaffold protein, that allows another level of chromatin interaction via LEDGF.

Menin is the product of the gene Multiple Endocrine Neoplasia Type 1 (*MEN1*) [25] and has context-specific functions. It is a bonafide tumor suppressor gene in neuroendocrine tissues [26]. On the other hand, Menin is absolutely necessary for the maintenance of aberrant gene expression by KMT2A-fusion proteins in the hematopoietic system [27, 28].

Here, we present evidence that while the SET domain of Kmt2a is not required for MN1-mediated transformation, Menin plays a major role in sustaining the MN1-driven leukemic program. Together with our previous findings of a requirement for *Kmt2a*, our data suggest that MN1 drives a leukemic program mediated by the Menin/Kmt2a complex, and that disrupting this complex represents a new therapeutic strategy in MN1-driven AML.

## Material and methods

For primer sequences, antibodies, and detailed experimental procedures, please refer to the Supplementary Methods.

### *Kmt2a* SET, *Men1* KO mice

*Kmt2a* SET germline knockout [29] and *Men1* [22] conditional KO mice have been published elsewhere.

### Cell lines and cultures

Human cell lines were purchased from the Deutsche Sammlung von Mikroorganismen und Zellkulturen (DSMZ, Germany) or the American Type Culture Collection (ATCC, US). UCSD-AML1 [*MN1-ETV6*] were maintained in RMPI-1640 (Corning®, Corning, NY, USA) supplemented with 20% fetal bovine serum (FBS) (Life Technologies, Carlsbad, CA, USA), 50 U/ML Penicillin–Streptomycin (P/S) (Life Technologies, Carlsbad, CA, USA) and 20 ng/mL human IL-6 and 40 ng/mL human GM-CSF (PreproTech Inc., Rocky Hill, NJ, USA). MV4;11 [*KMT2A-AFF1*], Molm14 [*KMT2A-MLLT3*], THP1 [*KMT2A-MLLT3*], OCI-AML3 [*NPM1c*], and KASUMI-1 cells *[RUNX1-RUNX1T1*] were maintained in RMPI-1640 supplemented with 10% FBS and 50 U/ml P/S. 293T cells were maintained in DMEM supplemented with 10% FBS and 50 U/ml P/S.

All cells were cultured in a humidified incubator at 37 °C in 5% CO_2_.

### Generation of murine MN1-driven leukemia

All experiments were conducted in accord with the principles and procedures outlined in the Internal Animal Care and Use Committee.

The MN1 cDNA was a gift from Ellen C. Zwarthoff (Erasmus University, Rotterdam, the Netherlands) and was cloned in the MSCV-IRES-GFP (MN1-GFP) plasmid. Kmt2a-Mllt3 and Hoxa9-Meis1 vectors were described earlier [30]. Ecotropic retroviral vectors containing murine MN1-IRES-GFP, Kmt2a-Mllt3-IRES-GFP, Hoxa9-IRES-GFP, Meis1-IRES-puro, and Cre-IRES-tdTomato (Cre) were generated by cotransfection of 293T cells. Lin^–^Sca^−^1^–^cKit^+^CD34^+^FcγR^lo^ (CMP) cells were transduced with the respective retroviral supernatants and maintained with supplemental cytokines. Hoxa9-IRES-GFP + Meis1-IRES-puro transduced cells were plated in puromycin 24 h after transduction. Two to three days after transduction, GFP^+^ cells were sorted and transduced with Cre. Two days after Cre-transduction, GFP^+^/tdTomato^+^ cells were sorted and transplanted into syngeneic irradiated recipients. For secondary transplants, blast colonies were allowed to grow out for 2–3 days. Leukemic cells were transduced with Cre and transplanted. For serial transplantation, blast colonies were allowed to grow out for 2–3 days and cells were directly transplanted into secondary recipients.

### Colony assays for murine MN1-driven AML

For colony assays, sorted transduced leukemia cells were plated in methylcellulose M3234 (STEMCELL^TM^ technologies, Vancouver, CANADA) supplemented with 10 ng/ml murine IL3 and IL6 and 20 ng/ml murine SCF (Preprotech Inc, Rocky Hill, NJ, USA), 10% P/S and IMDM (Corning®, Corning, NY, USA) at 1000–2000 cells per plate in duplicate and replated weekly at 500 cells/plate. *Men1* deletion was verified by PCR at each replating.

### Apoptosis assays of murine MN1-driven leukemic cells

For the flow cytometric assessment of apoptosis, cells were resuspended in Annexin buffer (10 mM HEPES, 150 mM NaCl, 5 mM KCl, 5 mM MgCl_2,_ 1.8 mM CaCl_2_ in water), stained with Annexin V (APC) (Biolegend®, San Diego, CA, USA), and DAPI for 15 min and analyzed by flow cytometry. Apoptotic cells were defined as Annexin V^+^ only cells and dead cells were defined as Annexin V^+^/DAPI^+^.

### Menin/Kmt2a inhibition in murine MN1-driven AML

MN1-driven leukemic cells (*Men1*^*wt*^ background) or control cells were plated in M3234 (STEMCELL^TM^ technologies, Vancouver, CANADA) supplemented with 10 ng/ml murine IL3 and IL6 and 20 ng/ml murine SCF (Preprotech Inc, Rocky Hill, NJ, USA 10% P/S and IMDM (Corning®, Corning, NY, USA) at a concentration of 500 cells per plate, in duplicates. VTP50469 (Syndax®) or DMSO was added at the indicated concentration. Every 5 days, colonies were scored based upon morphology and total cell numbers counted. Cells were then replated at the same concentration in fresh methylcellulose with fresh drug.

### Menin/KMT2A inhibition in human AML

Cells were plated in duplicates and VTP50469 (Syndax®) or DMSO control was added at the indicated concentration. Cell growth and viability were determined by trypan blue exclusion staining every 3 days and cells were replated in fresh media with fresh drug at equal densities for 16 days.

### UCSD-AML1 Xenograft model

NSGS (NOD-scid IL2Rgnull-3/GM/SF) were obtained from Jackson laboratories® and maintained under specific pathogen free conditions. Two million UCSD-AML1 cells were resuspended in PBS (Life Technologies, Carlsbad, CA, USA) and injected into the tail vein. Two weeks after the transplantation, mice were placed on VTP50469 0.1% chow or control chow. Myeloblasts were detected in peripheral blood, bone marrow and spleen after staining with a combination of anti-human CD45 (Alexa 700) and anti-mouse CD45 (FITC) antibodies. A first cohort was treated with VTP50269 for 25 days and sacrificed at day 50 to assess for leukemic burden. A second cohort was treated with continuous VTP50269 until animals reached a humanly defined survival endpoint.

### RNA extraction, qPCR and RNA-Seq

*Men1*^*−/−*^ and *Men1*^*wt*^ MN1-driven leukemic cells isolated from three independent moribund mice each were thawed and plated in M3234 methylcellulose. For compound experiments, MN1-driven leukemia cells were plated in the presence of the indicated concentration(s) of VTP50469 or DMSO control. After one methylcellulose replating, cell pellets were resuspended in QIAGEN RLT buffer (Hilden, Germany) with 2-Mercaptoethanol (1:100) and frozen at −80 °C. For extraction of RNA, samples were warmed to room temperature and extracted using the RNeasy mini kit from QIAGEN (Hilden, Germany) according to manufacturer instructions. Briefly, gDNA was removed by a column, the flow through was mixed 1:1 with 70% ethanol, the RNA was bound to a second column and washed with ethanol. RNA was eluted in water and quantified using a NanoDrop® spectrophotometer (Thermo Fisher Scientific, Waltham, MA, USA). For qPCR, RNA was reverse transcribed using qScript cDNA SuperMix (Quantabio) per the manufacturer’s instruction. qPCR was performed using PowerUp SYBR Green (Appliedbiosystems). RNA-Seq libraries were prepared from PolyA selected mRNA (eukaryotic), and multiplexed sequencing was conducted on Illumina HiSeq Sequencers to a read depth of 20–30 million per sample.

### RNA-seq analysis

Raw Fastq files were aligned using STAR [31] against reference Mus musculus GRCm38. All samples read-counts were quantified by Kallisto [32] (version 0.45.0). Output from Kallisto was then directly imported into DESeq2 [33] in order to detect differentially expressed genes (DEG). DEGs were deemed as genes with False Discovery Rate < 0.05. All analysis was carried using R, version 3.6.3.

Gene Set Enrichment Analysis (GSEA) was carried out using GSEA standalone software (Version 4.03) and “GSEAPreranked” tool [34]. “Stat” field from the output of DESeq2 was used as ranked list input for GSEA software. Gene lists in the “Gene sets database” were constructed from Heuser et al. [16] (“MN1 vs. Gr-1+/CD11b+” in Supplementary Table S2), Riedel et al. [19] (“downregulated in *Mll1*^*−/−*^ versus *Mll1*^*f/f*^*”* in Supplementary Table S3) and Krivtsov et al. [35] (“MOLM13_ChIP_Menin_VTP_Dn_150” and “MOLM13_ChIP_DOT1L_VTP_Dn_150” in Supplementary Table S5) (Supplementary Table S1). We selected “classic” as our parameter for enrichment score as suggested by the GSEA software instruction manual. RNA-Seq data is accessible in GEO: GSE150151.

### Statistical analysis

Statistical analysis of colony and cell numbers, apoptosis, percent expression of markers associated with differentiation, percent expression on GFP^+^ in bone marrow and peripheral blood was performed using the unpaired two-tailed Student’s *t*-tests or 2-way Anova (multiple parameters). Statistical analysis of survival was carried out using Kaplan Meyer estimates (Prism 8 software). The frequency of LIC was calculated using the ELDA software [36]. A *p* value of <0.05 was considered statistically significant.

## Results

### The histone methyltransferase activity of Kmt2a is dispensable to MN1-mediated transformation

We previously found that deletion of *Kmt2a* in MN1-driven leukemia led to a downregulation of the MN1-driven leukemic program [19]. The C-terminal SET domain of KMT2A mediates mono-, di- and trimethylation of lysine 4 on histone 3. H3K4me at *Hox* gene promoters is associated with active transcription [21, 37], although the exact function of H3K4me in gene expression is still a matter of debate [38]. In normal hematopoiesis, the SET domain was shown to be dispensable for Kmt2a function [39]. Nevertheless, peptide inhibitors of proteins that interact with KMT2A C-terminus such as WDR5 are reported to have preclinical activity in *KMT2a*-r AML [40]. Therefore, understanding whether the SET domain plays a role in MN1-driven leukemia could have future therapeutic implications.

In order to investigate a role for Kmt2a SET domain, we compared the ability of MN1 to transform bone marrow progenitors of mice with a constitutive homozygous deletion of the SET domain (*Kmt2a SET*^*−/−*^) with their wild type litter mates (*Kmt2a SET*^*wt*^). While homozygous deletion of *Kmt2a* is embryonically lethal, *Kmt2a SET*^*−/−*^ animals develop normally despite decreased expression of certain *Hox* genes (associated with a locus specific decrease in H3K4me1) [29]. Furthermore, they survive into adulthood and present no hematopoietic defects [39].

**Fig. 1:**
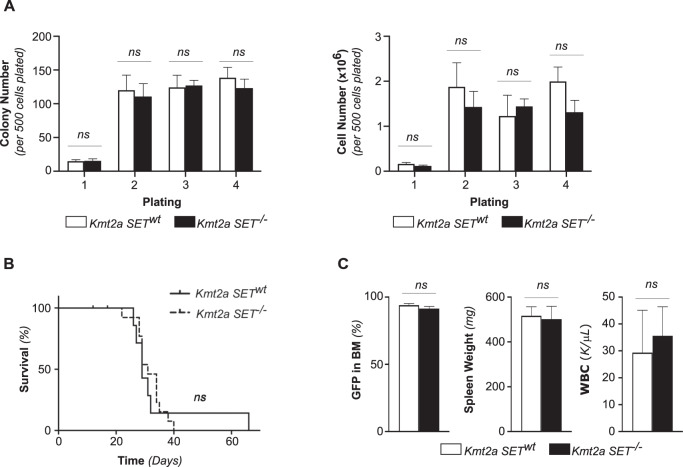
MN1-driven leukemia can be established in the absence of Kmt2a SET domain. **A** Serial replating of in vitro MN1-transformed *Kmt2a SET*^*−/−*^ and *Kmt2a SET*^*wt*^ CMP. Colony numbers (left) and cell numbers (right) per 500 cells plated in duplicate. Error bars represent mean ± SEM of three biological replicates over two independent experiments. ns = non-significant, unpaired double-sided *t*-test. **B** Survival of in vitro MN1-transformed *Kmt2a SET*^*−/−*^ (*n* = 13) and *Kmt2a SET*^*wt*^ (*n* = 7) CMP recipients. Two independent experiments. *p* = *ns*, Log rank (Mantel–Cox) test. **C** Leukemia burden at the time of sacrifice as measured by percentage of GFP^+^ cells in the bone marrow (left), spleen weights (middle) and total white blood cell count (WBC, right). Error bars represent mean ± SEM for *n* = 7 *Kmt2a SET*^*wt*^, *n* = 11 *Kmt2a SET*^*−/−*^. *p* = *ns*, unpaired double-sided *t*-test.

We expressed human MN1 using a retroviral vector (MN1-GFP) in murine CMPs. GFP^+^ sorted cells were then plated in methylcellulose to assess their serial replating capacity in vitro. We found no difference in the number or the size of colonies between MN1-transformed CMP established from a *Kmt2a SET*^*wt*^ or *Kmt2a SET*^*−/−*^ animals (Fig. 1A).

In parallel, GFP^+^ sorted cells were injected into sublethally irradiated syngeneic recipients to investigate their ability to establish leukemia in vivo. MN1-transformed *Kmt2a SET*^*−/−*^ CMP were able to induce leukemia with the same latency as wild type controls (median survival of 31 days in *Kmt2a SET*^−/−^ versus 29 days in controls, *p* = ns) (Fig. 1B). At the time of death, animals in both groups showed similar leukemic burden, as estimated by the percentage of GFP^+^ cells in the bone marrow, elevated white blood count and spleen weight (Fig. 1C).

Thus, the SET domain of Kmt2a (and therefore the corresponding histone methyltransferase activity) is not required for the establishment of MN1-driven leukemia in vitro or in vivo.

### Deletion of *Men1* impacts colony forming potential in vitro and decreases the number of leukemia initiating cells in vivo

**Fig. 2:**
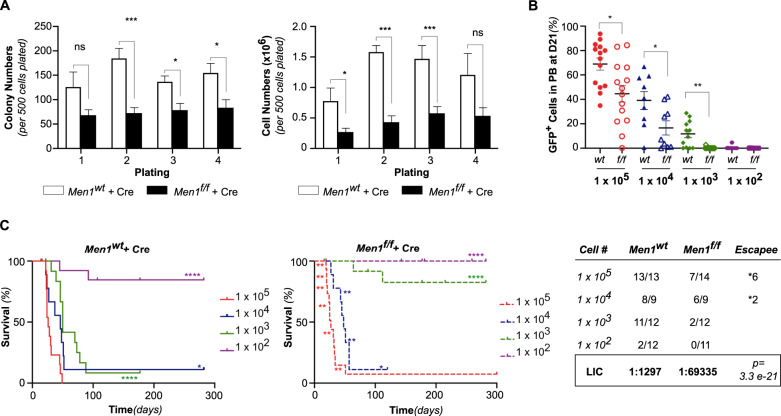
Loss of *Men*1 decreases the frequency of LIC. **A** Serial replating of in vivo transformed *Men1*^*f/f*^ and *Men1*^*wt*^ MN1-driven AML transduced with Cre. Colony numbers (left panel) and cell number (right panel) per 500 cells plated in duplicates. Cumulative data from three independent experiments. Error bars represent mean ± SEM of biological replicates (*n* = 3 *Men1*^*wt*^ and *n* = 5 *Men1*^*f/f*^ MN1-driven AML). ***p < 0.0005, *p < 0.05, *ns* = non-significant, unpaired double-sided t-test. **B** Peripheral blood (PB) engraftment at D21 post-transplantation. Percentage of GFP^+^ in PB (*Men1*^*wt*^ + Cre: 1 × 10^5^ *n* = 4, 1 × 10^4^ *n* = 9, 1 × 10^3^ *n* = 12, 1 × 10^2^ *n* = 12; *Men1*^*f/f*^ + Cre: 1 × 10^5^ *n* = 5, 1 × 10^4^ *n* = 9, 1 × 10^3^ *n* = 12, 1 × 10^2^ *n* = 12). Cumulative data from two independent experiments, using three distinct *Men1*^*f/f*^ MN1-driven AML donors. Error bars represent mean ± SEM. *p < 0.05, **p < 0.005, unpaired double-sided *t*-test. **C** Survival curves (left) and determination the LIC frequency using ELDA (right) [36]. of recipients of 1 × 10^5^ (*n* = 4), 1 × 10^4^ (*n* = 9), 1 × 10^3^ (*n* = 12), 1 × 10^2^ (*n* = 12) *Men1*^*wt*^ or *Men1*^*f/f*^ MN1-driven leukemic cells transduced with Cre. Cumulative data from two independent experiments. *p < 0.05, ****p < 0.0001, Log rank (Mantel–Cox) test.

Menin was found to be essential for transformation and maintenance of *KMT2A*-rearranged leukemias [27, 28] and our previous findings suggest that endogenous Kmt2a is required for MN1-driven leukemia [19]. Interestingly, the Menin/KMT2A interaction has also been found to be required in other subtypes of leukemia such as those with *NPM1c* [41] and *C/EBPα* mutations [42, 43]. Therefore, we next investigated whether the Kmt2a N-terminal interaction partner Menin was required in MN1-driven leukemia. We established in vivo MN1-driven leukemia on a conditional *Men1*^f/f^ background and utilized retroviral expression of *Cre*-recombinase (MSCV-*Cre*-tdTomato) to excise *Men1* alleles. *Men1*^*wt*^ MN1-driven AML cells transduced with *Cre* served as control (*Men1*^*wt*^ + *Cre*). In vitro, *Men1*^*f/f*^ + *Cre* cells formed consistently fewer colonies compared to *Men1*^*wt*^ + *Cre* cells (Fig. 2A). However, loss of *Men1* did not abolish colony forming ability over four replatings in MN1-driven murine AML.

Leukemia-initiating cells (LIC) are defined as cells capable of giving rise to human AML in immunocompromised mice [44] but constitute only a small subset of the whole leukemia population. To determine if the changes in colony forming ability upon loss of *Men1* were secondary to a decrease in the number of LIC, we transplanted Cre-transduced *Men1*^*f/f*^ and *Men*^*wt*^ MN1-driven leukemic cells at limiting dilutions. We found that at 1 × 10^5^, 1 × 10^4^ and 1 × 10^3^ *Men1*^*f/f*^ + *Cre* transplanted mice had a significantly lower leukemic burden on day 21 compared to *Men1*^*wt*^ + *Cre* (Fig. 2B). Given the highly aggressive nature, rapid growth and high percentage of LICs in this model, the difference in day 21 leukemic burden did not translate into a difference in disease latency in the *Men1*^*f/f*^ + *Cre* group at the 1 × 10^5^ and 1 × 10^4^ cell dose level (Fig. 2C and Supplementary Fig. S1A–C). However, we did note that a substantial number of *Men1*^*f/f*^ + *Cre* mice succumbed to leukemia that had failed to rearrange both alleles, implying strong selective pressure against loss of *Men1* (Supplementary Fig. S1D, E). Remarkably, at the 1 × 10^3^ cell dose level, loss of *Men1* severely impaired the ability of MN1-driven AML cells to establish leukemia (*p* < 0.0001 Fig. 2C). Overall, the frequency of LIC was significantly lower in *Men1*^*−/−*^ MN1-driven AML (1:69335 (IC95 [1:127285–1:37768])) than in *Men1*^wt^ MN1-driven AML (1:1297 (IC95 [1:2684–1:627])), *p* = *3.3e-21* (Fig. 2C*)*.

### *Men1*^−/−^ MN1-driven leukemic cells exhaust in vivo

**Fig. 3:**
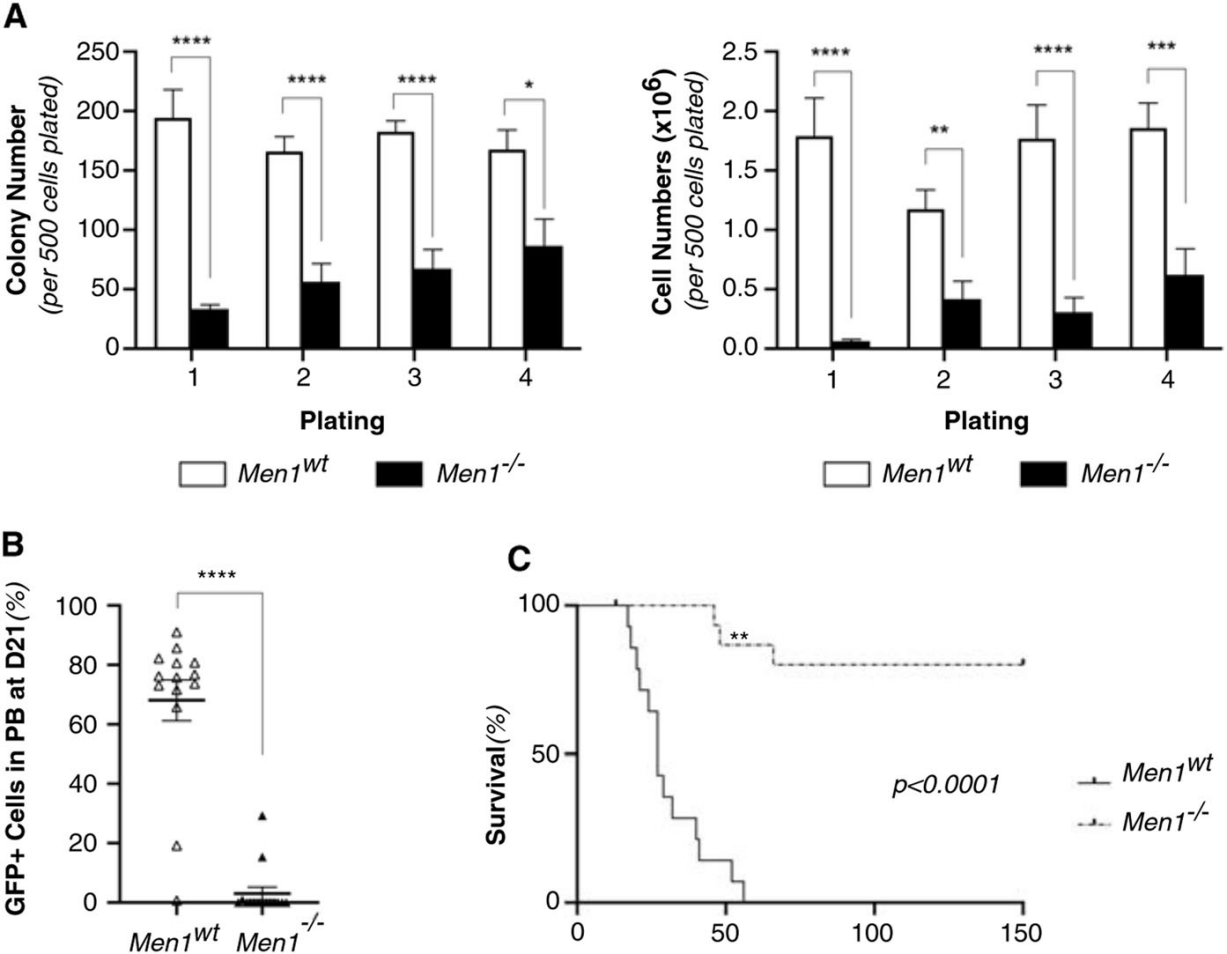
*Men1*^−/−^ MN1-driven leukemic cells exhaust in vivo. **A** Serial replating of *Men1*^*wt*^ and *Men1*^*−/−*^ MN1-driven AML cells isolated from moribund recipients. Colony and cell numbers per 500 cells plated in duplicates. Cumulative data from two independent experiments. Error bars represent mean ± SEM of biological replicates (*n* = 5 *Men1*^*wt*^ and *n* = 6 *Men1*^*−/−*^). *p < 0.05, **p < 0.005, ***p < 0.0005, ****p < 0.00005, unpaired double-sided *t*-test. **B** Peripheral blood (PB) engraftment at D21 post-transplantation. Percentage of GFP^+^ in PB in recipients of 50,000 *Men1*^*wt*^ and *Men1*^*−/−*^ MN1-driven AML cells (*n* = 15 animals for each condition over two independent experiments). Error bars represent mean ± SEM. ****p < 0.0001, unpaired double-sided *t*-test. **C** Survival of secondary recipients of 50,000 *Men1*^*wt*^ and *Men1*^*−/−*^ MN1-driven AML cells isolated from moribund recipients. *n* = 15 animals for each condition over two independent experiments. *p* < 0.0001, Log rank (Mantel–Cox) test. ** Signifies failure to properly re-arrange both allele (*Men1*^*f/-*^ leukemia).

We next analyzed *Men1*^*−/−*^ MN1-driven AML isolated from moribund animals in more detail. In vitro, *Men1*^*−/−*^ MN1-driven AML cells presented a profound impairment in both colony formation and cell growth (Fig. 3A, S2A). Annexin V staining revealed increases in apoptosis (Supplementary Fig. S2B). To assess if differences were also observed in vivo, we transplanted *Men1*^*−/−*^ MN1-driven AML cells isolated from five distinct moribund animals (and matched controls) into secondary irradiated recipients. At D21 post transplantation, we found a significant decrease in peripheral blood leukemic burden in the *Men1*^*−/−*^ versus *Men1*^*wt*^ group (*p* < 0.0001) (Fig. 3B). Furthermore, *Men1*^*−/−*^ MN1-driven AML cells failed to propagate leukemia in most recipients (13/15), with a failure to rearrange both alleles in one of the animals *(*Fig. 3C and Supplementary Fig. S2C, D*)*. This resulted in a dramatic improvement in survival compared to *Men1*^*wt*^ control (Fig. 3C*, p* < 0.0001).

### *Men1*^*−/−*^ AML exhibits loss of the MN1-driven leukemic program

We next investigated the transcriptional consequences of deletion of *Men1* in MN1-driven AML. RNA sequencing was performed on *Men1*^*−/−*^ and *Men1*^*wt*^ cells isolated from moribund animals after brief in vitro culture.

**Fig. 4:**
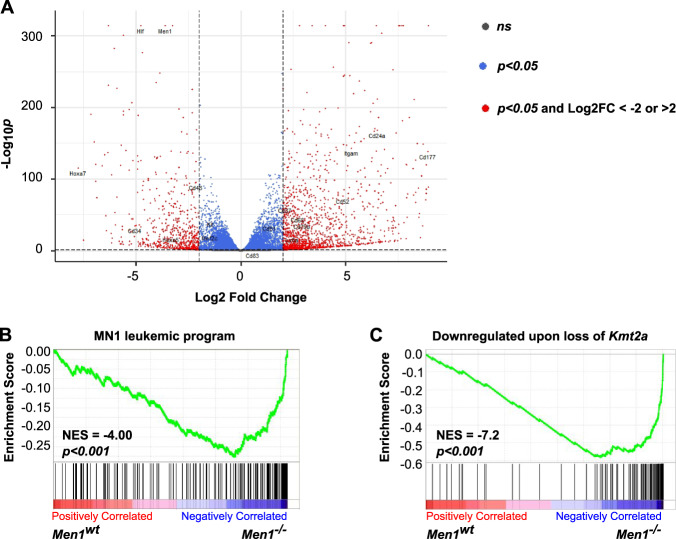
Loss of *Men1* downregulates the MN1 leukemic program. *Men1*^*wt*^ and *Men1*^*−/−*^ MN1-driven AML cells were isolated from moribund recipients and plated in methylcellulose for 7 days before RNA extraction. *n* = 3 biological replicates for each condition. **A** Volcano plot representing differentially expressed genes in in *Men1*^*−/−*^ versus *Men1*^*wt*^ MN1-driven AML cells. **B** GSEA showing downregulation of the MN1 leukemic program (Heuser et al. [16]) in *Men1*^*−/−*^ versus *Men1*^*wt*^ AML cells isolated from moribund recipients, after 7 days in methylcellulose. **C** GSEA showing enrichment of genes downregulated by loss of *Kmt2a* is MN1-driven leukemia (Riedel et al. [19]) in *Men1*^*−/−*^ versus *Men1*^*wt*^ MN1-driven leukemic cells isolated from moribund recipients, after 7 days in methylcellulose.

Analysis of differentially expressed genes (DE) in *Men1*^*−/−*^ versus *Men1*^wt^ MN1 driven leukemic cells revealed downregulation of 276 genes (Log2 Fold change (FC) < −2, *padj* < 0.05) and upregulation 645 of genes (Log2 FC > 2, *padj* < 0.05) (Fig. 4A, Supplementary Table S2). Gene Set Enrichment Analysis (GSEA) revealed downregulation of the MN1-driven leukemic gene expression program in *Men1*^*−/−*^ versus *Men1*^*wt*^ leukemias, a program previously defined by Heuser et al. [16] (Supplementary Table S1) as a group of genes that are upregulated in MN1-driven leukemic cells versus normal bone marrow (Fig. 4B). Moreover, genes that we had previously found to be downregulated upon *Kmt2a* deletion [19] (Supplementary Table S1) in this model were strongly enriched in *Men1*^*−/−*^ versus *Men1*^*wt*^ MN1-driven AML (Fig. 4C). We also interrogated a gene set defined as genes bound by Menin which exhibit displacement of Menin and transcriptional downregulation upon pharmacologic inhibition of the Menin/KMT2A interaction in a human *KMT2A-MLLT3* leukemia cell line [35]. We found this gene set enriched in *Men1*^*−/−*^ versus *Men1*^*wt*^ MN1-driven AML (Supplementary Fig. S3A). In addition, genes bound by the histone methyltransferase DOT1L and downregulated upon Menin inhibition in *KMT2A-MLLT3* leukemia [35] were found to be enriched in *Men1*^*−/−*^ versus *Men1*^*wt*^ MN1-driven AML (Supplementary Fig. S3B). We previously demonstrated a role for DOT1L in maintaining transcription of the MN1-leukemogenic program defined by Heuser et al. [16, 19]. Taken together, these data are consistent with a core transcriptional program that is regulated by Menin across several AML subtypes. *Men1*^*−/−*^ MN1-driven AML cells are unable to sustain this core leukemogenic gene expression program necessary for long-term maintenance, leading to cell death in vitro and engraftment failure in vivo.

### Inhibition of the Menin/Kmt2a interaction is an effective therapeutic strategy in MN1-driven AML

**Fig. 5:**
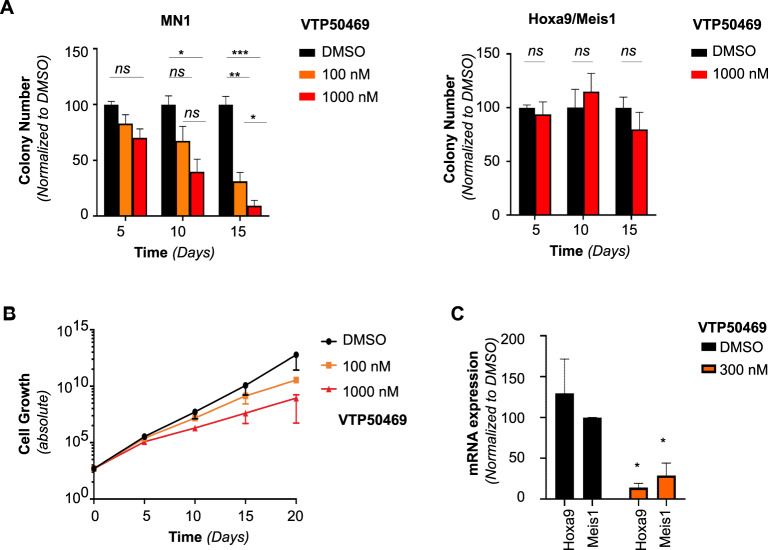
A small molecule inhibitor of the Menin/Kmt2a interaction decreases cell growth in MN1-driven murine AML. **A** Serial replating of in vivo-transformed *Men1*^*wt*^ MN1-driven AML cells, or Hoxa9/Meis1 driven negative control AML cells with dimethyl sulfoxide (DMSO), VTP50469 at the indicated concentrations. Error bars represent mean ± SEM of two experimental repeats. *p < 0.05, **p < 0.005, ***p < 0.0005, *ns* = non-significant, unpaired double-sided *t*-test. **B** Cell growth shown as fold-expansion over serial replating of in vivo-transformed *Men1*^*wt*^ MN1-driven AML with DMSO, 100 nM or 1000 nM VTP50469. Error bars represent mean ± SEM of two experimental repeats. **C** qPCR analysis of Hoxa9 and Meis1 expression in MN1-driven AML cells with DMSO or 300 nM VTP50469. Error bars represent mean ± SEM of three biological replicates. *p < 0.05, 2-way Anova.

VTP50469 is a potent and specific inhibitor of the Menin/KMT2A interaction with demonstrated activity against *KMT2A*-fusion leukemia [35] and *NPM1*-mutated leukemia, where it targets wild-type KMT2A [41, 45]. In MN1-driven murine AML, we found that the gene expression program downregulated upon loss of *Men1* and loss of *Kmt2a* significantly overlapped, suggesting that Menin might mediate the effect of Kmt2a. Therefore, we investigated the effects of pharmacological inhibition of the Menin/Kmt2a interaction in murine MN1-driven AML. We found that inhibition of the Menin/Kmt2a interaction decreased colony formation (Fig. 5A) and cell growth (Supplementary Fig. S4A) in methylcellulose in MN1-driven AML cells, but not in control cells driven by retroviral overexpression of Hoxa9 and Meis1. Fifty percent growth inhibition occurred at similar dose levels for MN1-driven AML and Kmt2a-Mllt3 driven murine AML, although it should be noted that in the Kmt2a-Mllt3 model, responses occurred earlier, and higher doses resulted in considerably more profound inhibition (Fig. 5B and Supplementary Fig. S4B, C). In addition, VTP50369 treatment resulted in downregulation of the key downstream target Meis1 [35, 45] (Fig. 5C and Supplementary Fig. S4D*)*. As previously reported for *KMT2A*-r cells, VTP50469 also caused upregulation of the differentiation marker Cd11b (Supplementary Fig. S4E, F), although cells did not undergo terminal differentiation as demonstrated by the largely maintained blast morphology on cytospin (Supplementary Fig. S4G).

**Fig. 6:**
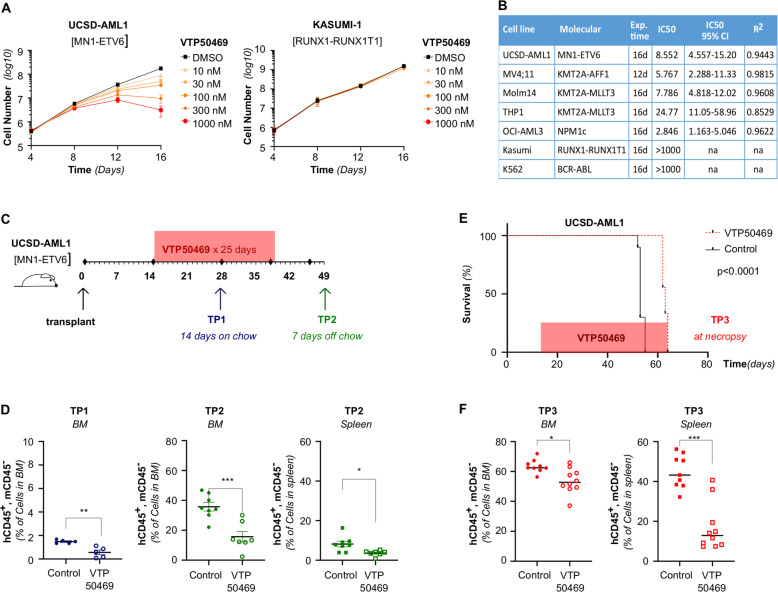
The Menin/KMT2A interaction is a therapeutic target in human MN1- driven AML. **A** Cell growth of UCSD-AML1 cells [*MN1-ETV6*] and Kasumi-1 cells [*RUNX1-RUNXT1*] plated with DMSO or VTP50469 at the indicated doses shown as absolute cell numbers over 16 days of treatment. Error bars represent mean ± SEM of two biological replicates. **B** Dose response (IC50) of UCSD-AML1 and control cell lines (*KMT2A-*r: MV4;11, Molm14, THP1, *NPM1c*: *OCI-AML3*, negative control *RUNX1-RUNXT1*: Kasumi, *BCR-ABL*: K562). Error bars represent mean ± SEM of three technical replicates. **C** Schematic representing the UCSD-AML1 xenograft experimental set up. NSGS mice were transplanted at D0. VTP50469 chow was started at D14 post-transplant for a total of 25 days. Leukemia burden was assessed at Time Point 1 (TP1), after 14 days on chow, at D28 post-transplant and at Time Point 2 (TP2), 7 days after discontinuing chow, at D47 post-transplant. **D** Leukemia burden in the bone marrow at TP1 (left panel), as well as bone marrow (middle panel) and the spleen (right panel) at TP2, in VTP50469 versus control treated mice assessed by the percentage of human CD45^+^, murine CD45^−^ cells. Error bars represent mean ± SEM of biological replicates (*n* = 5 animals per condition, unpaired double-sided *t*-test.). **E** Survival of mice NSG mice transplanted with UCSD-AML1 cells and treated with VTP50469 or control chow. VTP50469 chow was started at D14 post-transplant and continued throughout the experiment. Kaplan–Meier estimate, *p* < 0.0001 *n* = 10 animals per condition. **F** Leukemia burden in the bone marrow (left) and the spleen (right) at necropsy in VTP50469 versus control treated mice assessed by the percentage of human CD45^+^, murine CD45^−^ cells. Error bars represent mean ± SEM, *n* = 10 animals per condition, unpaired double-sided *t*-test.

We next wanted to investigate the effect of VTP50469 in human MN1-driven AML. To test a human AML in which MN1 overexpression was likely to be a founding mutation, we used UCSD-AML1 cells, which carry a *t*(12;22) translocation. In AML cells with this rearrangement, most or all of the coding sequence of *MN1* translocates to the C-terminal of *ETV6*, resulting in very high expression of MN1 either as a full-length protein or as an MN1-ETV6 fusion protein [4, 46]. Disruption of the Menin/KMT2A interaction with VTP50469 produced a time- and dose-dependent decrease in proliferation in vitro. This was not the case upon treatment of KASUMI-1 [*RUNX1-RUNXT1*] cells, a cell line characterized by low *HOXA9* and *MEIS1* expression (Fig. 6A). Overall, UCSD-AML1 cells displayed sensitivity to VTP50469 with an IC50 that was higher than the highly sensitive MV4;11 [*KMT2A-AFF1*] and OCI-AML3 [*NPM1c*] cell lines, similar to Molm-14 [*KMT2A-MLLT3*], and lower than THP1 [*KMT2A-MLLT3*] (Fig. 6B and Supplementary Fig. S5). Of note, MV4;11 responded much earlier and much more profoundly at higher doses compared to UCSD-AML1 and the other *KMT2A*-rearranged cell lines (Fig. 6B and Supplementary Fig. S5).

In order to determine efficacy of VTP50469 in vivo, we established a UCSD-AML1 xenograft model. On D0, NSGS mice were injected with 2 × 10^6^ UCSD-AML1 cells. After 14 days, animals were switched onto to VTP50469-containing or control chow (Fig. 6C). We performed a first assessment of leukemia burden at D28 post-transplant/D14 of treatment (TP1). At TP1, we found a small but significant difference between control and drug-treated mice (Fig. 6D). Chow was discontinued after 25 days and mice were sacrificed on D47 post-transplant, 7 days after discontinuing the chow. At TP2, we observed a significant decrease in both the bone marrow and the spleen leukemia burden in VTP50469-treated mice (Fig. 6D*)*. In a second independent experiment, animals were transplanted and treatment was initiated as detailed above, but VTP-50469 or control chow were continued for the remainder of the experiment. Sustained VTP-50469 treatment induced a modest but significant increase of survival in addition to lower leukemic burden (Fig. 6F, G). Overall, pharmacologic inhibition of the Menin/KMT2A interaction decreased cell growth of both murine and human MN1-driven leukemia, with efficacy in vivo. These data underscore the value of targeting the Menin/KMT2A interaction in MN1-driven AML.

## Discussion

MN1-driven leukemia is characterized by a CMP-like gene expression program that includes high expression of *Hoxa9* and *Meis1* [16], known target genes of Kmt2a-fusion proteins but also of wild type Kmt2a. We previously established that aberrant gene expression in MN1-driven leukemia was under the control of wild type Kmt2a [19]. As Kmt2a is involved in chromatin regulation through distinct functional domains and interaction partners, we investigated here which are relevant to MN1-mediated transformation.

Using a murine conditional knockout model, we found that loss of *Men1* specifically impacts the LIC frequency in MN1-driven AML. In primary recipients, a decrease in the number of LIC was illustrated by the inability of *Men1*^*f/f*^ MN1 leukemic cells transduced with Cre to engraft at lower cell number (<1 × 10^4^ cells). In secondary recipients, the decrease in LIC resulted in an almost complete exhaustion of *Men1*^*−/−*^ MN1-driven leukemia cells in vivo. Furthermore, GSEA revealed that a set of genes that we had previously found to be downregulated upon loss of *Kmt2a* in MN1-driven leukemia was enriched in *Men1*^*−/−*^ versus *Men1*^*wt*^ MN1-driven AML cells. Menin-dependent genes in MN1-AML also contained a core transcriptional program that was found to be dependent on Menin in other subtypes of AML, including those with KMT2A rearrangements and NPM1c [35]. The observation that loss of *Men1* and loss of *Kmt2a* have similar effects on gene expression suggests that such effect is mediated through the Menin/Kmt2a interaction. Indeed, pharmacologic inhibition of the Menin/Kmt2a interaction decreased colony formation and leukemia burden in models of murine and human MN1-driven AML in vivo and in vitro, respectively.

Menin interacts with the N-terminus of both wild type KMT2A [47] and KMT2A fusion proteins [28]. In *KMT2A*-rearranged leukemias, the transcriptional consequences of *Men1* deletion/Menin/KMT2A inhibition mirror inactivation of the fusion, not inactivation of the *KMT2A* wild type allele [48]. The unmutated *KMT2A* allele is largely dispensable for KMT2A-fusion mediated leukemogenesis [48, 49]. Thus, Menin/KMT2A inhibition in *KMT2A*-fusion leukemias acts via disruption of the fusion complex, not the KMT2A complex. In contrast, Menin/KMT2A inhibition is effective in disrupting KMT2A dependent transcription in leukemia models that exhibit a dependency on KMT2A by preventing recruitment of the Menin/wild type KMT2A complex to a subset of target genes [41, 45]. Interestingly, among the key canonical targets of KMT2A or KMT2A-fusions in these models, MEIS1, but not HOXA cluster gene expression was found to be directly dependent on Menin. This suggest that the observed downregulation of HOXA cluster gene expression in these and our models is likely an indirect effect mediated by the disruption of a HOXA/MEIS1 dependent auto-regulatory loop [50]. The strong overlap between genes downregulated upon inactivation of *Kmt2a* and inactivation of *Men1* supports that the effect of Menin/Kmt2a inhibition in MN1-driven leukemias is a result of disrupting the Kmt2a complex. Overall, our data confirms that inhibition of the Menin/KMT2A interaction is a therapeutic strategy in the absence of *KMT2A*-fusion proteins in selected AML subtypes.

The observation that *KMT2A*-rearranged leukemias are critically dependent on Menin [27] and more particularly the interaction between Menin and KMT2A [28] has prompted the development of small molecule inhibitors of this protein-protein interaction [51]. Several generations of improved inhibitors have demonstrated specific inhibition of leukemia cell growth in vitro, induction of differentiation as well as downregulation of both *HOXA9* and *MEIS1* expression [51–53]. Furthermore, small molecule inhibitors of the Menin/KMT2A interaction have been shown to induce complete remission as single agents in PDX models [35, 54]. In steady state hematopoiesis, Menin was found to be dispensable, as acute loss of *Men1* only causes minor cytopenias [55] and does not affect the frequency of hematopoietic stem cells (HSC) and other progenitors. Still, *Men1*^*−/−*^ HSCs were at a disadvantage compared to *Men1*^*wt*^ HSCs in repopulating secondary recipients after bone marrow ablation. Thus, this raises the concern that targeting the Menin/KMT2A interaction in leukemias that depend on wild type KMT2A could also negatively impact normal hematopoiesis. Prolonged treatment of non-leukemic mice with inhibitors of the Menin/Kmt2a interaction did not reveal any significant effect on normal hematopoiesis [35, 54]. Similarly, we found that in a xenograft model of MN1-driven leukemia, VTP50469 was well tolerated at doses that decreased leukemic burden. Furthermore, no toxicity of a therapeutically effective dose was observed in a murine model and in PDX of NPM1c AML [45, 54]. This suggests a therapeutic window for inhibition of the Menin/KMT2A interaction in leukemias that rely on KMT2A.

While the exact contribution of MN1 to leukemogenesis remains unclear, high levels of expression of MN1 consistently correlate with poor prognosis; whether it is found in the absence of any karyotypic anomalies [6–8] or when associated with rearrangement of the MN1 locus [4, 56]. In addition, high levels of MN1 have been found in a subgroup of AML patients with dismal prognosis, those with complex karyotype or monosomy 5 and 7 [9]. Overall, for patients with high MN1 expression, current AML therapy remains inefficient. The data described here demonstrate that Menin specifically regulates a rare but key population of MN1-driven leukemic cells, the LIC. LIC have shown decreased drug sensitivity compared to the bulk of the leukemia population [57] and have been directly linked to relapse [58]. Thus, inhibition of Menin might allow to specifically target the subpopulation of leukemia cells resistant to conventional chemotherapies.

## Supplementary information

Supplemental Material

Supplementary table 1 revised

Supplementary table 2
