# Supplementary material for: Menin is necessary for long term maintenance of meningioma-1 driven leukemia
Source: Leukemia. 2021 Feb 4;35(5):1405–17. doi: 10.1038/s41375-021-01146-z (PMC8102197; doi:10.1038/s41375-021-01146-z)
Supplement: Supplementary file 1 — Supplemental Material [file 41375_2021_1146_MOESM1_ESM.pdf]

SUPPLEMENTAL MATERIALS

- **Supplementary Figures**
- **List of supplementary tables**
- **Supplementary Materials and Methods**

Supplementary Figure 1: Complete loss of *Men1* is counterselected *in vivo*

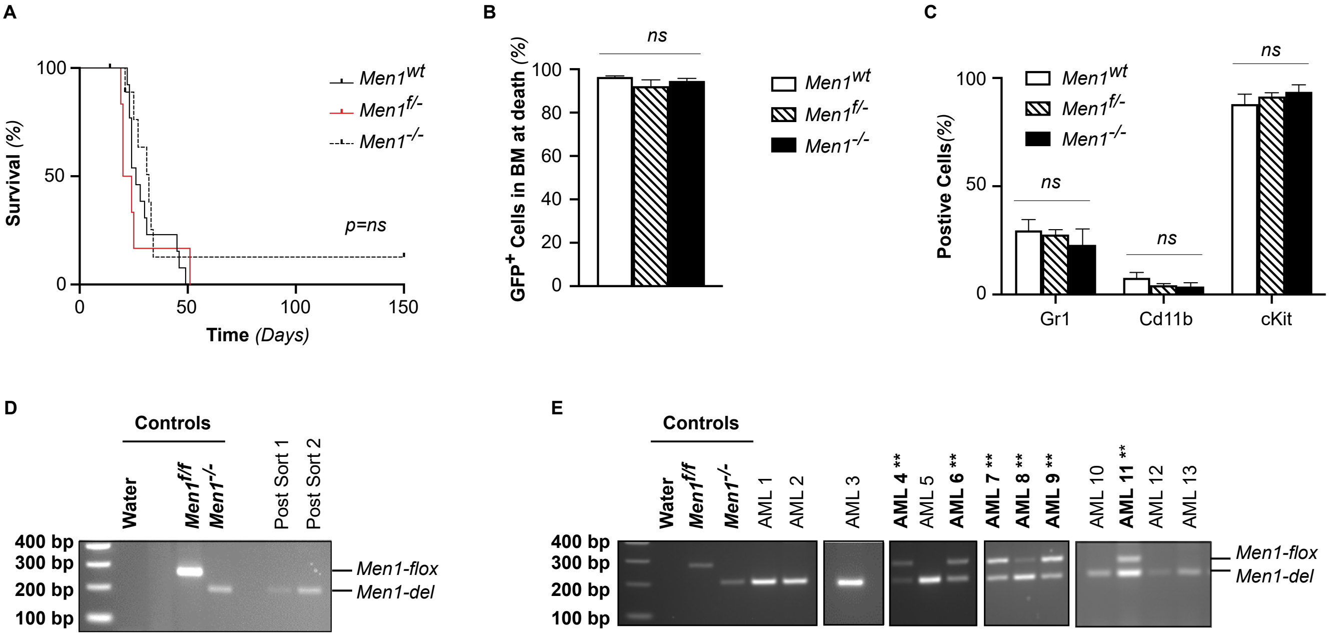

Supplementary Figure 1: Complete loss of *Men1* is counter-selected *in vivo*

A. Survival of recipients of 100,000 *Men1*$^{f/f}$ and *Men1*^{wt} MN1-driven leukemic cells isolated from moribund mice and transduced with *Cre* presented by *Men1* status (n= 13 *Men1*^{wt} MN1-driven AML, *n*=6 *Men1*$^{f/-}$ MN1-driven AML, n=7 *Men1*$^{-/-}$ MN1-driven AML*).* Cumulative data from 2 independent experiments. *ns=non-significant*, Log rank (Mantel-Cox) test.

B. Leukemia burden at the time of sacrifice recipients of 100,000 *Men1*$^{f/f}$ and *Men1*^{wt} MN1 -driven leukemic cells isolated from moribund mice and transduced with *Cre*. Percentage of GFP+ cells in the bone marrow (n= 13 *Men1*^{wt} MN1-driven AML*, n*=6 *Men1*$^{f/-}$ MN1-driven AML, n=7 *Men1*$^{-/-}$ MN1-driven AML*).* Cumulative data from 2 independent experiments. Error bars represent mean +/- SEM. *p=ns*, unpaired double-sided t-test.

C. Leukemia phenotype at the time of sacrifice of recipients of 100,000 *Men1*$^{f/f}$ and *Men1*^{wt} MN1-driven leukemic cells transduced with Cre. Expression of Gr1, Cd11b and cKit by flow cytometry. (n= 13 *Men1*^{wt} MN1-driven AML*, n*=6 *Men1*$^{f/-}$ MN1-driven AML, n=7*, Men1*$^{-/-}$ MN1-driven AML*).* Cumulative data from 2 independent experiments. Error bars represent mean +/- SEM. *p=ns*, unpaired double-sided t-test.

D. Representative DNA genotyping gel confirming deletion of *Men1* in *Men1*$^{f/f}$ MN1-driven AML cells transduced with Cre on the day of transplant.

E. DNA genotyping gels of bone marrow isolated from recipients in the *Men1*$^{f/f}$ + *Cre* group at the time of sacrifice*.* ** denotates *Men1* heterozygous deletion and signifies outgrowth of *Men1*$^{f/f}$ *or Men1*$^{f/-}$ MN1-driven AML cells.

Genotyping PCR for *Men1*$^{f/f}$ + *Cre* animals were not run on the same gel, but each gel included a size marker and the appropriate controls. Individual gels are indicated through dividing lines.

Supplementary Figure 2 : *Men1-/-* MN1-driven leukemic cells exhaust *in vivo*.

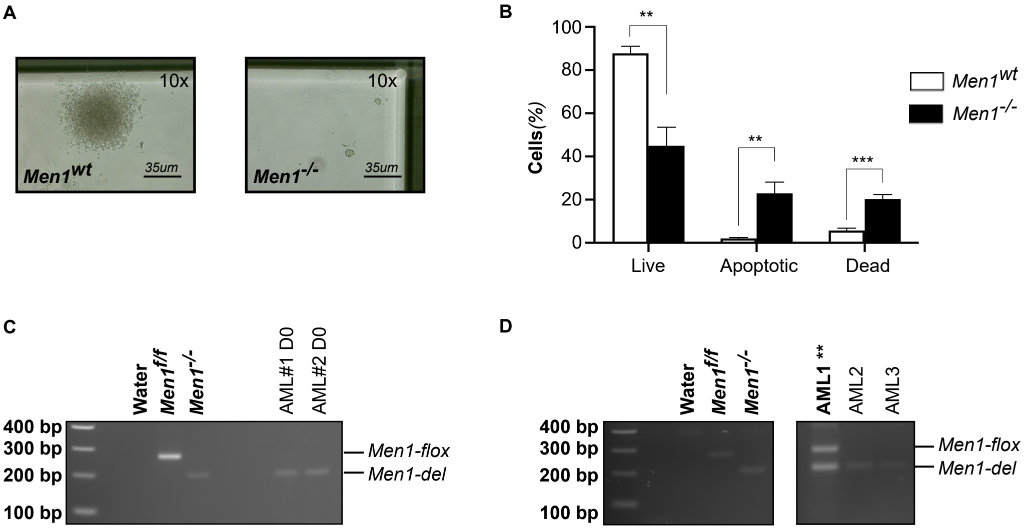

Supplementary Figure 2: *Men1$^{-/-}$* MN1-driven leukemic cells exhaust *in vivo*.

A. Methylcellulose colony morphology of *Men1^{wt}* and *Men1$^{-/-}$* MN1-driven AML cells isolated from moribund recipients after 7 days in culture.

B. Annexin V and DAPI staining of *Men1^{wt}* and *Men1$^{-/-}$* MN1-driven AML cells isolated from moribund recipients after 7 days in methylcellulose. Apoptotic cells are defined as Annexin V positive only, dead cells are defined as Annexin V and DAPI double positive. Cumulative data from 2 independent experiments. Error bars represent mean +/- SEM of biological replicates (n= 5 *Men1^{wt}* and n=6 *Men1$^{-/-}$*). **p<0.005, ***p<0.0005. unpaired double-sided t-test.

C. Representative DNA genotyping gel confirming deletion of *Men1* in *Men1$^{-/-}$* MN1-driven AML cells isolated from moribund recipients on the day of the transplant.

D. DNA genotyping gels of bone marrow isolated from the 3 secondary recipients of *Men1$^{-/-}$* MN1-driven AML cells that succumbed of their disease. ** denotes *Men1* heterozygous deletion and signifies outgrowth of *Men1$^{f/f}$ or Men1$^{f/-}$* MN1-driven AML cells.

Supplementary Figure 3: A shared core regulatory program is controlled by Menin in AML

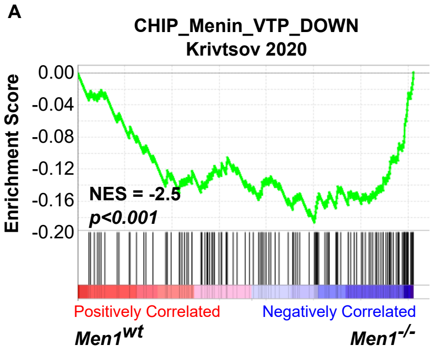

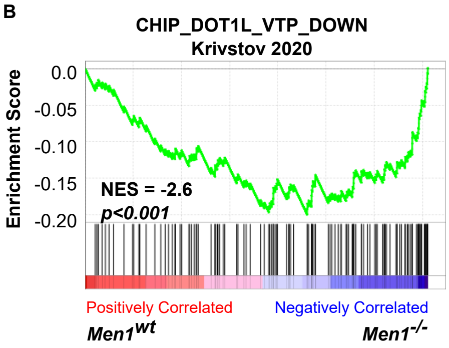

Supplementary Figure 3: A shared core transcription program is controlled by Menin in AML.

A. GSEA showing enrichment of genes bound by Menin which exhibit displacement of Menin and are downregulated upon Menin inhibition in Molm13 cells (KMT2A-MLLT3, *Krivtsov et al.(35)*) enriched in *Men1*$^{-/-}$ versus *Men1*^{wt} MN1-driven leukemic cells isolated from moribund recipients, after 7 days in methylcellulose.

B. GSEA showing enrichment of genes bound by DOT1L and downregulated upon Menin inhibition in Molm13 cells (KMT2A-MLLT3, *Krivtsov et al.(35)*) enriched in *Men1*$^{-/-}$ versus *Men1*^{wt} MN1-driven leukemic cells isolated from moribund recipients, after 7 days in methylcellulose.

Supplementary Figure 4: Cell growth, Meis1 expression and differentiation of MN1-AML and control murine leukemias

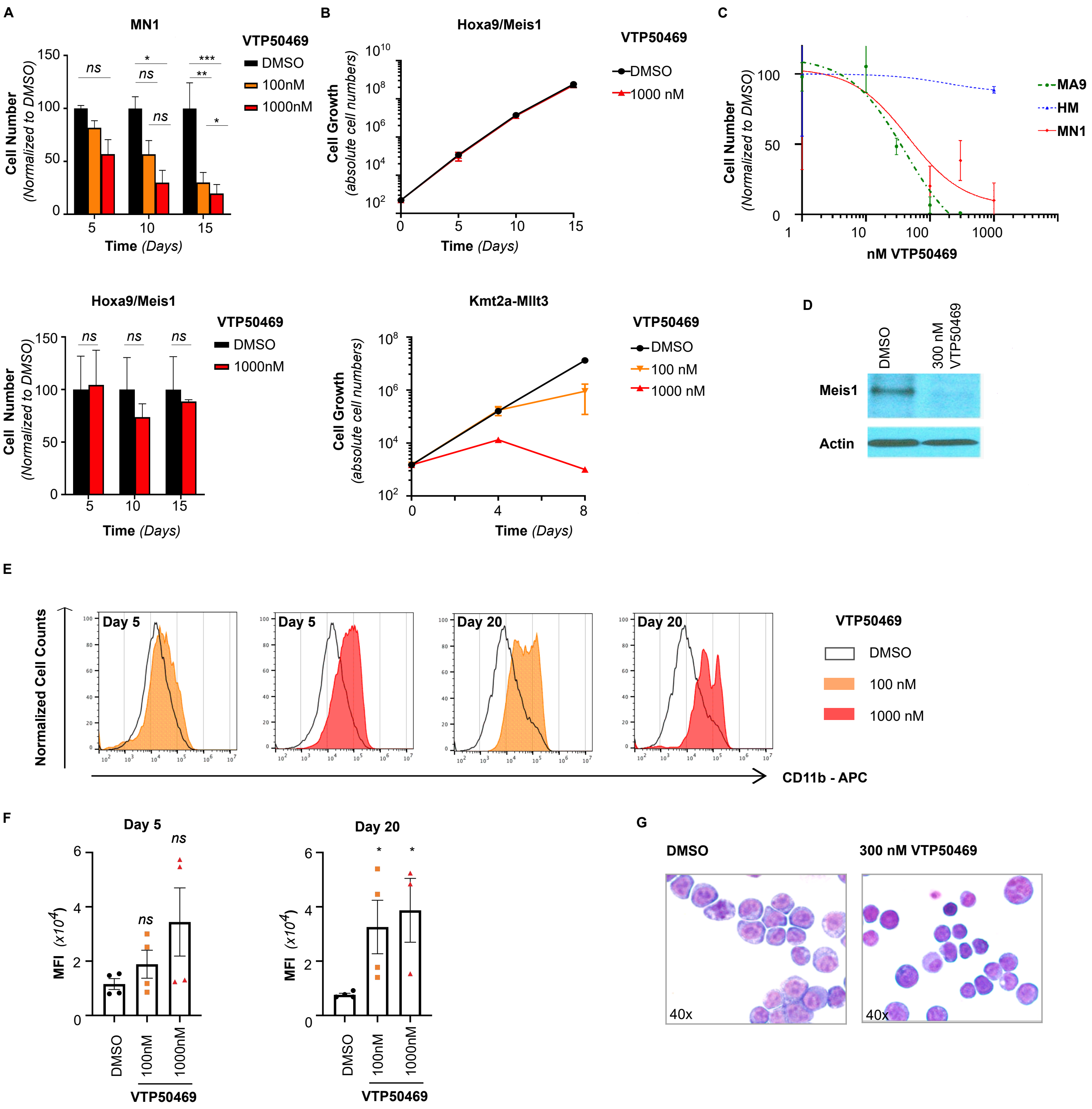

Supplementary Figure 4: Cell growth, Meis1 expression and differentiation of MN1-AML and control murine leukemias

A. Cell numbers (normalized to dimethyl sulfoxide (DMSO)) from serial replating of *in vivo*-transformed *Men1*^{wt} MN1-driven AML cells, or Hoxa9/Meis1 driven negative control AML cells with DMSO, VTP50469 at the indicated concentrations. Error bars represent mean +/- SEM of 2 experimental repeats. * p<0.05, ***p<0.005,* *** *p<0.0005*, *ns=non-significant*, unpaired double-sided t-test.

B. Cell growth shown as absolute cell numbers over serial replating of cells transformed with Hoxa9/Meis1 (top panel) or Kmt2a-Mllt3 (bottom panel) with DMSO, 100nM or 1000nM VTP50469. Error bars represent mean +/- SEM of 2 experimental repeats (Hoxa9/Meis1) or 3 technical repeats (Kmt2a-Mllt3).

C. Dose-response of *in vivo*-transformed *Men1*^{wt} MN1-driven AML, Kmt2a-Mllt3 -driven positive control (MLL-Af9, “MA9”) and Hoxa9/Meis1 (“HM”) driven negative control AML cells to VTP50469. Of note, response for MN1 and Hoxa9/Meis1 plateaued and was assessed on day 12, while the Kmt2a-Mllt3 response plateaued and was assessed on day 8 (see Figure S3). Error bars represent mean +/- SEM of 2 experimental repeats (Hoxa9/Meis1) or 3 technical repeats (Kmt2a-Mllt3).

D. Protein expression of Meis1 in MN1-driven AML cells with DMSO or 300nM VTP50469 by Western Blotting.

E. Representative flow plots for Cd11b expression by flow cytometry after 5 and 20 days of treatment of *in vivo*-transformed *Men1*^{wt} MN1-driven AML with DMSO, 100nM or 1000nM VTP50469.

F. Analysis of Cd11b expression by flow cytometry after 5 days (up) and 20 days (down) of treatment of *in vivo*-transformed *Men1*^{wt} MN1-driven AML with DMSO, 100nM or 1000nM VTP50469. *ns=non-significant, * p<0.05*

G. Cytospin of MN1-driven AML cells treated with 300 nM VTP50469 or DMSO control.

Supplementary Figure 5: In vitro growth of cell lines exposed to VTP50469

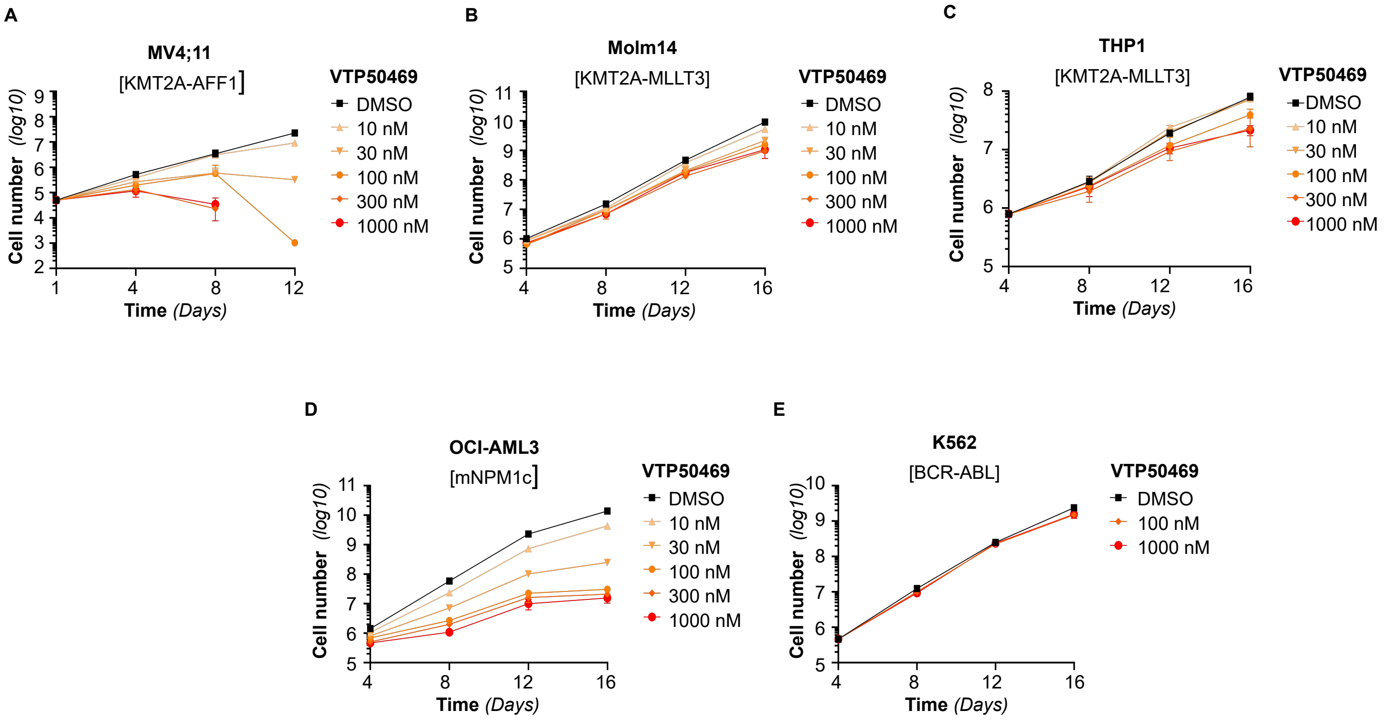

Supplementary Figure 5: Cell growth of human AML cell lines exposed to VTP50469

Cell growth of **(A)** MV4;11 [*KMT2A-AFF1*], **(B)** Molm14 [*KMT2A-MLLT3*], **(C)** THP1 [*KMT2A-MLLT3*], **(D)** OCI-AML3 [*NPM1c*], and **(E)** K562 [*BCR-ABL*] plated with DMSO or VTP50469 at the indicated doses shown as absolute cell numbers over 16 (or 12 for the rapidly responding MV4;11 cell line) days of treatment. Error bars represent mean +/- SEM of 2 biological replicates.

Supplementary table 1: Comparative gene lists used for GSEA analysis

- Supplied as separate excel file

Supplementary table 2: Differentially expressed genes in *Men1*$^{-/-}$ versus *Men1*^{wt} MN1-driven AML

- Supplied as separate excel file

SUPPLEMENTARY MATERIAL AND METHODS

Resource Table

**Antibodies**		
Rabbit polyclonal anti-MEIS1	Abcam	Cat # 19867, lot # GR3319572-1
Mouse polyclonal anti-actin	Milipore Sigma	Cat # MAB1501, RRID:AB_2223041
Goat anti mouse secondary H+L-HPR conjugated	BioRad	Cat# 170-6516, RRID:AB_11125547
Goat anti rabbit secondary H+L-HPR conjugated	BioRad	Cat# 170-6515, RRID:AB_11125142
CD3, Biotin, anti-mouse	Biolegend	Cat# 100304, RRID:AB_312669
CD4, Biotin, anti-mouse	Biolegend	Cat# 100404, RRID:AB_312689
CD8α, Biotin, anti-mouse	Biolegend	Clone 53-6.7, Cat# 100704, RRID:AB_312743
Gr1 (Ly6-G/Ly6-C), Biotin, anti-mouse	Biolegend	Clone RB6-8C5y, Cat# 108404, RRID:AB_313369
Gr1 (Ly6-G/Ly6-C), PeCy7, anti-mouse	Biolegend	Cat# 108416, RRID:AB_313381
MAC1, APC, anti-mouse	Biolegend	Clone M1/70, Cat# 101212, RRID:AB_312795
B220, Biotin, anti-mouse/human	Biolegend	Clone RA3-6B2, Cat# 103204, RRID:AB_312989
CD19, Biotin, anti-mouse	Biolegend	Clone 6D5, Cat# 115504, RRID:AB_313369
IL-7Rα (CD127), Biotin, anti-mouse	Biolegend	Clone A7R34, Cat# 135006, RRID:AB_2126118
Ter-119, Biotin, anti-mouse	Biolegend	Clone TER-119, Cat# 116204, RRID:AB_313705
Streptavadin, APC-Cy7	Biolegend	Cat# 405208
Ckit (CD117), Alexa Fluro 647, anti-mouse	Biolegend	Clone 2B8, Cat# 105818, RRID:AB_493474
Sca-1 (Ly6A), Pe-Cy7, anti-mouse	Invitrogen	Clone D7, Ref# 25-5981-82, RRID:AB_469669
FCγR, PE, anti-mouse	Biolegend	Cat# 101307, RRID:AB_312806
CD34, FITC, anti-mouse	Biolegend	Cat# 553733
Dynabeads M-280, Streptavidin	Invitrogen	Ref# 11206D
CD45, FITC, anti-mouse	BD Pharmingen	Clone 30-F11
CD45, Alexa Fluor 700, anti-human	BD Pharmingen	Clone: HI30

Annexin-APC	BD Biosciences	550474

**Recombinant DNA**		
MSCV-MN1-IRES-GFP	Bernt Lab (cDNA from Ellen Zwaarthof)	NA
MSCV-IRES-dTomato	Addgene	Plasmid #107229
MSCV-Cre-IRES-dTomato	Armstrong Lab	N/A
MSCV-HOXA9-IRES-GFP	Armstrong Lab	N/A
MSCV-Meis1-IRES-puro	Armstrong Lab	N/A
MSCV-KMT2A-MLLT3-IRES-GFP (MLL-AF9, MA9)	Armstrong Lab	N/A
**Cytokines**		
Recombinant Murine IL-3	PeproTech	Cat# 213-13
Recombinant Murine IL-6	PeproTech	Cat# 216-16
Recombinant Murine SCF	PeproTech	Cat# 250-03
Recombinant Murine TPO	PeproTech	Cat# 315-14
Recombinant Murine FLT3-Ligand	PeproTech	Cat# 250-31L
Recombinant human GM-CSF	PeproTech	Cat# 315-03
**Cell lines**		
UCSD-AML1 (female)	DSMZ	ACC691, RRID:CVCL_1853
293 (female)	ATCC	CRL-1573
**Cell Culture Reagents**		
RPMI-1640 Media	VWR	Cat# 10-040-CV
IMDM (Iscove's modif of DMEM)	VWR	Cat# 45000-366
DMEM	VWR	Cat# 45000-312
Methylcellulose M3234	Sigma	Cat# M7140
Fetal Bovine Serum	Life Technologies	Cat# 10438026
L-Glutamine	Life Technologies	Cat# 25030081
Penicillin-Streptomycin (10,000 U/mL)	Invitrogen	Cat# 15140122
Fugene 6 Transfection Reagent	VWR	Cat# PAE2692
OptiMEM	Thermo	Cat# 31985-062
Retronectin	Clontech Laboratories	Cat# T100B
Polyethylene glycol	Sigma	Cat# P4338
Trypan Blue Solution	Mediatech	Cat# MT25-900-CI
Phosphate Buffered Saline	Mediatech	Cat# MT21-031-CV
Lookout mycoplasma PCR detection kit	Sigma	Cat # MP0035-1KT
**Other Reagents**		
BD Pharmlyse	Fisher BD	Cat# 555899

**Chemicals**		
TritonX-100	VWR	Cat# 9002-93-1
Hydrochloric Acid	Fisher Scientific	Cat# A144
Sodium Chloride (NaCl)	Sigma Aldrich	Cat# S9888
NP-40 (IGEPAL)	Alfas Aesar	Cat# J61055
Sodium dodecyl sulfate (SDS)	Teknova	Cat# S0288
Sodium Deoxycholate	Alfas Aesar	Cat# J62288
Tris-HCl	Roche	Cat # 10812846001
Lithium Chloride (LiCl)	Alfas Aesar	Cat# 36217
Ethylenediaminetetraacetic acid (EDTA)	VWR	Cat# E1777
Sodium Bicarbonate ($NaHCO_3$)	Fisher Scientific	Cat# S233
**Kits**		
QiAquick PCR Purification Kit	Qiagen	Cat# 28106,
RNeasy Plus Mini Kit	Qiagen	Cat# 74136
qScript cDNA SuperMix	Quantabio	Cat #101414- 106 66162052
PowerUp SYBR Green Master Mix	Appliedbiosystems	Cat # A25742- 00843598
ZymoPURE II Plasmid Maxi Prep Kit	Zymo	Cat# 11-555B
Click-iT EdU Alexa Fluor™ 647 Flow Cytometry Assay Kit	Invitrogen	Cat# C10419
Qubit Protein Assay Kit	LifeTechnologies	Cat# Q33211
**Software and Algorithms**		
FlowJo (Flow cytometry)	Standard software	
Graphpad Prism	Standard software	Prism 8
Kallisto (RNA-Seq alignment)	(Bray et al., 2016)	Version 0.45.0
DESeq2 (DEG)	(Love et al., 2014)	Release 3.1
EnhancedVolcano (volcano plots)	Blighe K, Rana S, Lewis M (2020)	R package version 1.6.0, https://github.com/kevinblighe/EnhancedVolcano)
GSEA	(Subramanian et al., 2005)	Version 4.0

Virus Production

Ecotropic retroviral vectors (10 µg) containing murine MSCV-*MN1*-IRES-GFP or MSCV-*Cre*-IRES-dTom were co-transfected with delta psi packaging (10 µg) in 293T cells using FuGENE 6 (Promega, Madison, WI, USA) and Opti-MEM^{TM} (Life Technologies, Carlsbad, CA, USA). Supernatant containing viral particles was

collected 12, 36 and 72 hours after transfection. To concentrate the virus, polyethelene glycol was added to viral containing media 1:5 overnight at 4 °C, followed by centrifugation at 2500 rpm for 20 minutes at 4 °C. The concentrated virus was resuspended in PBS at a concentration of 10 µL of concentrated virus was equivalent to 1 mL of viral supernatant.

Generation of transformed murine cells and leukemia

Bone marrow cell suspensions were prepared by crushing leg bones in a mortar after removal of muscle and connective tissues. Red blood cells were lysed on ice using red blood cells lysis buffer BD Pharm Lyse^{TM} (BD Biosciences, Franklin Lakes, NJ, USA). Lineage depletion was performed by labeling bone marrow cell suspensions with a mixture of purified biotinylated monoclonal antibodies to CD3e, CD4, CD8a, CD19, B220, Gr-1, IL-7R and Ter-119. Lin^{+} cells were partially removed by 2 rounds of magnetic bead depletion with streptavidin conjugated Dynabeads (Thermo Fisher Scientifc, Waltham, MA, USA). Common Myeloid Progrenitors (CMP) cells were prepared by staining lineage depleted (Lin^{-}) cells with APC-Cy7 conjugated streptavidin and stained with c-Kit Alexa 647, Sca-1 PE-Cy7, CD34 FITC and FcRγ PE sorted for $Lin^{-}Sca^{-}1^{-}cKit^{+}CD34^{+}$FcRγ low. Sorted cells were pre-stimulated for 24 h with 10 ng/ml murine IL3 and IL6 and 20 ng/ml murine SCF, Flt3L and TPO (PeproTech, Rocky Hill, NJ, USA). Transduction was carried out on RetroNectin® (Takara Bio Inc., JAPAN) with in the presence of murine IL3, IL6, SCF, Flt3L and TPO in concentrations as above. Cells were subsequently maintained in M3234 methylcellulose (STEMCELL^{TM} technologies, Vancouver, CANADA) with 10 ng/ml murine IL3 and mIL6, 20 ng/ml mSCF, and 50 U/ml Penicillin/Streptomycin (Life Technologies, Carlsbad, CA, USA). After 2 days, GFP+ or GFP+/dTom+ cells were

sorted and either transplanted into mice or maintained in methylcellulose. Transplantations were performed into 6-week-old C57BL/6 female (Jax®) irradiated (650 RAD) at indicated numbers with the addition of 1x 10^5 of wild type C57BL/6 support bone marrow. Animals were irradiated in batched of 10, and mice were assigned to groups so that each batch was equally distributed among the treatment groups.

Western blotting and cytospin assays of murine MN1-driven leukemic cells

For Western blotting, whole protein lysates were lysed in RIPA (150mM sodium chloride, 50mM Tris pH 8, 1% NP-40, 0.5% sodium deoxycholate, 0.1% SDS) buffer with protease inhibitor for 15 minutes on ice. The lysed cells were centrifuged for 10 minutes at 15000 rpm in a standard benchtop centrifuge at 4°C. The supernatant was collected. For long-term storage whole protein lysates were kept at -20°C. For protein electrophoresis, histone or whole protein lysates were added to 4X LDS buffer and 10X reducing agent, and boiled at 85°C for 5 minutes. Proteins were separated on a 10% Bis-Tris gel and blotted on nitrocellulose membranes. Followed by 1 hour of blocking in 5% non-fat milk made with TBS-T. All antibodies were diluted into 5% non-fat milk made with TBS-T. αMeis1 was used at 1:1000, and αactin was used at 1:2000. Secondary antibodies were used at 1:10000. Proteins were visualized using Western Lightning Plus-ECL. For cytospins, 50,000 cells were washed twice in PBS, spun onto glass slides using the Thermo-Fisher Cytospin 4 at 800 rpm x 10 min, and stained for 30 sec each using DipQuick stain J0322A1-3 (Jorgensen Laboratories, Loveland, CO).

UCSD-AML1 Xenograft model

6-8 week old NSGS (NOD-scid IL2Rgnull-3/GM/SF) were obtained from Jackson laboratories® and maintained under specific pathogen free conditions. Mice were allowed to acclimatize for at least one week. Mice did not receive conditioning prior to transplantation, as our pilot experiments showed robust engraftment in most animals with and without prior conditioning. Two million UCSD-AML1 cells were resuspended in PBS (Life Technologies, Carlsbad, CA, USA) and injected into the tail vein. In experiment #1, 25 mice were transplanted. Two-weeks after the transplantation, mice were placed on VTP50469 0.1% chow or control chow. Although a very low level of leukemic cells can be detected in the peripheral blood of mice at day 14, pilot experiments showed that there is next to no correlation between peripheral blood leukemic burden (which is generally low in this model) and bone marrow or spleen leukemic burden, therefore, animals were not randomized based on d14 peripheral blood leukemic burden. Rather, mice were allocated based on cage (cage 1: control, cage 2: VTP50469, cage 3: control, cage 4: VTP50469, cage 5: spit up into two cages for control and VTP50469). 10 mice were sacrificed at TP1 (5 per group), the remainder was sacrificed at TP2. Myeloblasts were detected in peripheral blood, bone marrow and spleen after staining with a combination of anti-human CD45 (Alexa 700) and anti-mouse CD45 (FITC) antibodies. A second cohort of 20 mice was transplanted as described above and treated with continuous VTP50269 or control chow (allocation as above) until animals reached a humanly defined survival endpoint. For the UCSD survival xenograft experiment, the sample size was based on an anticipated mean survival of 60 days with a standard deviation of 10%, and an anticipated prolongation of survival by 20% with a similar standard deviation in treated animals. The sample size was chosen to have at least 80% power to detect a significance level of 0.05 using Lamorte's power calculations.

Primer sequences

Men1 genotyping primers

1R: TATCCAGCGATCACACACCCT

2R: GATGCTAAAGGGTCCTCCCTG

3F: CTTGGCTGGACGTAAACT

mHoxa9 qPCR primers

F: AGGCAAGGCCAGATTTGA

R: CCCGGCAGAACAATAACG

Meis1 qPCR primers

F: CACAGGAGACCCGACAATG

R: CATGTCCCCCGAGTTGAC
